# Sodium valproate improves sensorimotor gating deficit induced by sleep deprivation at low doses

**DOI:** 10.3906/sag-2011-229

**Published:** 2021-06-28

**Authors:** Muhammet TEKİN, Fatma Duygu KAYA-YERTUTANOL, Burcu ÇEVRELİ, Asil Ali ÖZDOĞRU, Hamza KULAKSIZ, Tayfun İ. UZBAY

**Affiliations:** 1 Applied Psychology Master’s Program, Institute of Health Sciences, Üsküdar University, İstanbul Turkey; 2 Department of Psychiatry, Faculty of Medicine, Üsküdar University, İstanbul Turkey; 3 Neuropsychopharmacology Practice and Research Center, Üsküdar University, İstanbul Turkey; 4 Department of Psychology, Faculty of Humanities and Social Sciences, Üsküdar University, İstanbul Turkey; 5 Neuroscience Master’s Program, Institute of Health Sciences, Üsküdar University, İstanbul Turkey; 6 Department of Medical Pharmacology, Faculty of Medicine, Üsküdar University, İstanbul Turkey

**Keywords:** Sleep deprivation, sodium valproate, prepulse inhibition, sensorimotor gating, psychosis, rat

## Abstract

**Background/aim:**

Sleep deprivation disrupts prepulse inhibition of acoustic startle reflex and can be used to mimic psychosis in experimental animals. On the other hand, it is also a model for other disorders of sensory processing, including migraine. This study aims to assess the effects of sodium valproate, a drug that is used in a variety of neuropsychiatric disorders, on normal and disrupted sensorimotor gating in rats.

**Materials and methods:**

Sixty-two Wistar albino rats were randomly distributed into 8 groups. Subchronic and intraperitoneal sodium valproate were administrated to the sleep-deprived and nonsleep-deprived rats by either 50–100 or 200 mg/kg/day. Prepulse inhibition test and locomotor activity test were performed. Sleep deprivation induced by the modified multiple platform method.

**Results:**

Sleep deprivation impaired prepulse inhibition, decreased startle amplitude, and increased locomotor activity. Sodium valproate did not significantly alter prepulse inhibition and locomotor activity in nonsleep-deprived and sleep-deprived groups. On the other hand, all doses decreased locomotor activity in drug-treated groups, and low dose improved sensorimotor gating and startle amplitude after sleep deprivation.

**Conclusion:**

Low-dose sodium valproate improves sleep deprivation-disrupted sensorimotor gating, and this finding may rationalize the use of sodium valproate in psychotic states and other sensory processing disorders. Dose-dependent effects of sodium valproate on sensorimotor gating should be investigated in detail.

## 1. Introduction

To model psychotic disorders in experimental animals, neurodevelopmental, pharmacological, lesion, and genetic manipulation methods are frequently utilized. However, there is still no animal model where psychosis can be reflected by its all aspects. Sleep deprivation (SD) may be a valid psychosis model due to its effects on behavioral, neurochemical, and neurophysiological parameters. 

It was reported that, with SD, cognitive, perceptual, and behavioral symptoms of psychosis in people and behavioral symptoms of psychosis in animals were observed [1,2]. Psychosis-like behaviors such as hyperactivity, irritability, aggression, perseverative behaviors, and increased response to psychostimulants are observed in rodents due to long-term SD [2,3]. Schizophrenia and SD seem to be causing similar functional connectivity anomalies [4–6] and neurophysiological [7–11] and neurochemical anomalies [12–16] in the brain. Moreover, the thalamic reticular nucleus (TRN), which is assumed to have a role in schizophrenia, is the place where the sleep spindles observed in electroencephalography are produced during NREM sleep, and evidence shows a decrease in the numbers and intensities of sleep spindles in nonmedicated schizophrenia patients, their first-degree relatives, and SD-applied healthy individuals [17–19]; these indicate possible common neurophysiological processes between SD and schizophrenia. For all these reasons, SD may be an ideal model to trigger a schizophrenia-like psychosis picture in experimental animals not only because it induces psychosis-like symptoms on the behavioral dimension but also because it probably partially imitates psychosis-related neurophysiological and neurochemical mechanisms. 

Furthermore, with SD, the prepulse inhibition (PPI) test is disrupted in humans and animals [20,21]. As known, PPI refers to inhibition of the startle response by a weaker stimulus that does not lead to startling and that comes before a strong sensory stimulus that leads to startling [22]. The phenomenon of PPI seen in all mammals is a relatively constant neurobiological marker [23], and it is used as a measurement of sensorimotor gating [24]. It is accepted that agents reversing the PPI that is disrupted in schizophrenia have therapeutic potential. Usage of SD as a psychosis model was proposed as it disrupts PPI in both humans and animals [25].

On the other hand, dysregulation of sensory processing is suggested in several neurological disorders such as migraine. Migraine is characterized by the central sensitization of the trigemino-thalamic pathway that results in a sensitivity to certain modalities of sensorial input [26], and somatosensory gating is thought to be altered [27,28]. Hence, SD is used as an experimental model for migraine [29] or for other sensory processing disorders such as mania [30].

This study aimed to investigate the effect of sodium valproate (SVA), a widely used antiepileptic/mood stabilizer (MS) drug in several neuropsychiatric disorders, on sensorimotor gating in sleep-deprived and nonsleep-deprived rats. The effects of SVA on post-SD sensorimotor gating have not been assessed before. Here, 72-h SD was used to mimic psychosis via disruption of prepulse inhibition, and effects of SVA, an add-on treatment in psychotic disorders, on this model were investigated by using the PPI test and locomotor activity test.

## 2. Materials and methods

### 2.1. Animals and laboratory

All experiments were performed at the Neuropsychopharmacology Application and Research Center (NPARC) of Üsküdar University. Sixty-two 14–16-week-old Wistar albino male rats were randomized into a total of eight groups including 4 groups without SD (nonsleep-deprivation; NSD) and 4 groups with SD. Two groups had 7 rats while others had 8 rats in each. Both the NSD and SD groups consisted of vehicle groups and SVA-administrated groups with doses of 50 mg/kg/day, 100 mg/kg/day, and 200 mg/kg/day. Injections were made intraperitoneally for 4 days 2 times a day as in the morning and the evening, and after the last injection in the morning of the 5th day, the experiments were started. 

Throughout the experiment, the animals were kept at a suitable room temperature (22 ± 2 °C), 40%–45% humidity, 12 h of light/dark cycles (light in the period of 07:00–19:00), and in a way that they would have access to food and water ad libitum. The subjects on which SD was applied were housed in a water tank for 72 h. The summary of the experimental protocol may be seen in Table 1. 

**Table 1:** Experiment protocol.

	Groups	Number of rats (n)	Hours	Day 1	Day 2	Day 3	Day 4	Day 5
NSD	Vehicle	8	09:00 17:00	S/HC S/HC	S/HCS/HC	S/HCS/HC	S/HCS/HC	S/HC-
	SVA 50 mg/kg/day	7	09:00 17:00	SVA/HCSVA/HC	SVA/HCSVA/HC	SVA/HCSVA/HC	SVA/HCSVA/HC	SVA/HC-
	SVA 100 mg/kg/day	8	09:00 17:00	SVA/HCSVA/HC	SVA/HCSVA/HC	SVA/HCSVA/HC	SVA/HCSVA/HC	SVA/HC-
	SVA 200 mg/kg/day	7	09:00 17:00	SVA/HCSVA/HC	SVA/HCSVA/HC	SVA/HCSVA/HC	SVA/HCSVA/HC	SVA/HC-
SD	Vehicle	8	09:00 17:00	S/HC S/HC	S/WTS/WT	S/WTS/WT	S/WTS/WT	S/WT -
	SVA 50 mg/kg/day	8	09:00 17:00	SVA/HCSVA/HC	SVA/WTSVA/WT	SVA/WTSVA/WT	SVA/WTSVA/WT	SVA/WT-
	SVA 100 mg/kg/day	8	09:00 17:00	SVA/HCSVA/HC	SVA/WTSVA/WT	SVA/WTSVA/WT	SVA/WTSVA/WT	SVA/WT-
	SVA 200 mg/kg/day	8	09:00 17:00	SVA/HCSVA/HC	SVA/WTSVA/WT	SVA/WTSVA/WT	SVA/WTSVA/WT	SVA/WT-

### 2.2. Ethical issues

This study was carried out in strict accordance with the recommendations in the Guide for the Care and Use of Laboratory Animals of the National Institutes of Health, USA, which was published and released in 1996. The protocol was approved by the Local Ethics Committee of Üsküdar University on June 23, 2016 with the approval number of 2016-13. 

### 2.3. Drug

This study used the 400 mg/4 mL intravenous form of SVA (Depakin®, Sanofi-Aventis, France) that is parenterally applied in humans, and each vial contained 400 mg of SVA. As the drug could be used intraperitoneally in experimental animals [31], it was applied in each injection by dissolving in 0.5 mL of saline. In the vehicle groups, each injection contained only 0.5 mL of saline.

### 2.4. Prepulse inhibition test

For measurement of the PPI of the acoustic startle reflex, an SR-LAB Startle Response System (San Diego Instruments, San Diego, California, USA) was used. The device for the PPI test consisted of four isolation cabinets, software, control unit, and animal enclosures. All animals were handled for 3 days prior to the start of the experiments to prevent anxiety, fear and/or stress of rats that could impair the startle response [32]. The rats were placed into the Plexiglas cylinders of the cabinets for 15 min for adaptation 24 h before the test. On the test day, the test protocol was applied. PPI% was defined as a decrease in the amplitude of the startle reflex in the presence of a prepulse stimulus and calculated for each of the three different prepulse intensities by using the following formula: , where ρ+ is the startle amplitude with the prepulse, and ρ- is the startle amplitude without the prepulse. After each session, the chambers were cleaned with ethanol. In order to prevent the habituation of the animal, intersession intervals were at least 1 week [33]. 

#### 2.4.1. Test protocol

The animals that were handled by the researcher for the first 3 days (D1-3) before the test were kept in the measurement boxes without stimulation for 15 min for their getting used to the device on D4, and the PPI test was ran on D5. This test starts with the habituation step lasting for 5 min where no stimulation is given, and only the background sound is present. In the following step, five 120 dB auditory stimuli are given. The blocks of stimuli are then consecutively repeated eight times. In each of these blocks, five different auditory stimuli with randomly changing orders are applied with random intervals (varying between 10 and 30 s). These are:

1. 40-ms 120 dB auditory stimulus,

2. 40-ms 120 dB auditory stimulus 100 ms after a 20-ms basal + 4 dB prestimulus,

3. 40-ms 120 dB auditory stimulus 100 ms after a 20-ms basal + 8 dB prestimulus,

4. 40-ms 120 dB auditory stimulus 100 ms after a 20-ms basal + 16 dB prestimulus,

5. Background sound only (this stimulus is for controlling responses arising from the rat’s movement within the cage).

Finally, 5 startle stimuli given at the beginning of the measurement are applied again at random intervals (10–30 s), and the startle response is assessed. The entire protocol lasts approximately 26 min.

### 2.5. Locomotor activity test

The locomotor activity (LMA) test was carried out in an open-field activity monitoring system with nine sound-insulated chambers with dimensions of 40 × 40 × 30 cm (MAY 9908, Commat Ltd., Ankara, Turkey). The activity of the subjects was monitored and analyzed by an automated video-tracking device and software (Noldus, Ethovision v3.1, Netherlands). LMA was measured by determining the amount of “total distance moved” for the 30-minute-long test period.

### 2.6. Modified multiple platform technique

SD induction by this technique is dependent on that, when rats that are forced to stand on small platforms placed into a water-filled tank enter REM sleep, their muscles lose tone, their tails contact the water, and thus, they awaken from the REM sleep. While it is usually reported that this mainly induces SD unique to the REM stage of sleep, studies have stated that it also affects the NREM stage at rates reaching about 40% [34]. Upon understanding that this technique induces anxiety in animals, it was proposed that this anxiety could be reduced by testing animals living in the same cage together [35] and allowing them to move freely among multiple platforms placed into the tank [36]. This way, the technique was modified in a way to allow the animals to be tested together and by moving. In different studies in the literature, tanks were used in different dimensions [37,38]. In this study, to trigger SD, a Plexiglas water tank with dimensions of 125 × 43 × 45 cm was used. Inside the tank, 14 platforms with a diameter of 6.5 cm and height of 16 cm each were placed so that they would have a distance of 10 cm from each other. The water tank was filled with water up to the last 1 cm of the platforms, that is, at a height of 15 cm. The water temperature was adjusted at 23 ± 1 °C. The tank was covered with grills that allowed access to water and food like in the cages. The animals of an entire group were put into the tank simultaneously. 

### 2.7. Statistical analysis

The statistical analysis of the data was performed using the PSPP Statistical Analysis Software 1.2.0 (GNU, Boston, MA, USA) and RStudio 1.3.1093 (RStudio, PBC, Boston, MA, USA). As the hypothesis of normal distribution (the Shapiro–Wilk test) was rejected, nonparametric tests were used for the statistical analysis. The effects of drug treatment and SD on PPI% were analyzed using the F1F1 function of R Program nparLD package, a nonparametric test for mixed analysis of variance with repeated measures, where drug treatment or SD was the whole-plot factor and prepulse intensity was the repeated one. We reported ANOVA-type statistics (ATS). The Kruskal–Wallis test was used to compare the basal mean PPI% among all groups, startle amplitude, and total distance moved values of SVA-treated NSD or SD groups. The Mann–Whitney U test was used to compare startle amplitude and total distance moved values of the vehicle groups. A value of p < 0.05 was considered statistically significant.

## 3. Results

A nonsignificant difference between the basal mean PPI% of all groups indicates that the groups are similar in terms of the basal mean PPI% [X2 (7) = .66, p = .99 > .05]. All data including the basal mean PPI%, mean PPI%, startle amplitude, and total distance moved are shown in Table 2. The weights of the subjects are shown in Table 3.

**Table 2:** Basal mean PPI%, mean PPI%, startle amplitude, and total distance moved values of all groups.

Groups		Number of rats (n)	Basal mean PPI% ± s.d.74 dB prepulse	Mean PPI% ± s.d.			Startle amplitude ± s.d.	Total distance moved (cm) ± s.d.
				78 dB prepulse	86 dB prepulse			
NSD	Vehicle	8	50.37 ± 14.74	51.86 ± 16.40	69.14 ± 11.70	78.55 ± 10.55	78.65 ± 17.53	656.26 ± 303.33
	SVA 50 mg/kg/day	7	52.14 ± 15.72	59.86 ± 11.45	74.14 ± 6.73	82.32 ± 6.24	71.61 ± 20.33	438.76 ± 140.24
	SVA 100 mg/kg/day	8	49.25 ± 15.12	56.27 ± 25.66	78.37 ± 9.93	85.46 ± 8.12	122.53 ± 94.33	435.41 ± 178.73
	SVA 200 mg/kg/day	7	52.14 ± 10.31	57.67 ± 12.88	71.50 ± 10.76	75.30 ± 12.61	67.45 ± 57.39	446.73 ± 156.23
SD	Vehicle	8	50.00 ± 13.86	34.07 ± 22.79	41.58 ± 22.50	62.84 ± 27.46	63.88 ± 61.44	982.17 ± 173.69
	SVA 50 mg/kg/day	8	52.87 ± 13.41	36.60 ± 26.88	50.38 ± 25.94	69.90 ± 10.00	146.40 ± 105.42	688.97 ± 184.10
	SVA 100 mg/kg/day	8	54.25 ± 13.18	17.99 ± 44.99	21.64 ± 31.95	56.59 ± 17.69	43.88 ± 33.63	796.07 ± 219.35
	SVA 200 mg/kg/day	8	52.75 ± 16.10	16.68 ± 47.08	30.18 ± 28.77	45.89 ± 37.21	56.26 ± 92.89	806.39 ± 233.95

**Table 3:** Weights of subjects.

Groups		Number of rats (n)	Weight ± s.d.
NSD	Vehicle	8	323.50 ± 25.24
	SVA 50 mg/kg/day	7	309.42 ± 48.65
	SVA 100 mg/kg/day	8	307.25 ± 43.03
	SVA 200 mg/kg/day	7	360.00 ± 42.11
SD	Vehicle	8	299.50 ± 39.47
	SVA 50 mg/kg/day	8	351.87 ± 40.96
	SVA 100 mg/kg/day	8	295.12 ± 19.19
	SVA 200 mg/kg/day	8	319.50 ± 37.02

### 3.1. SVA effects in NSD groups

**Figure 1:**
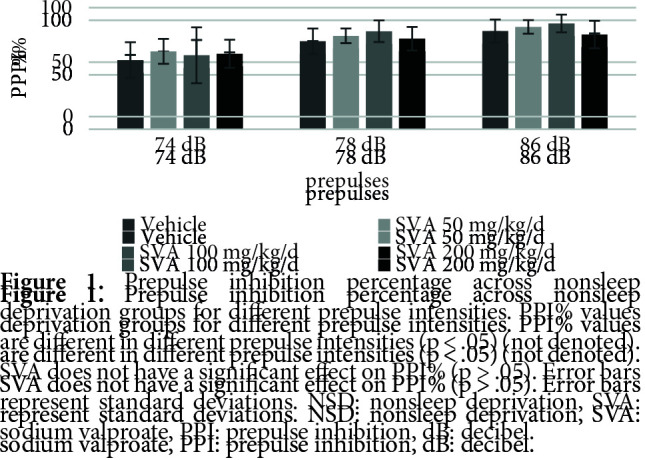
Prepulse inhibition percentage across nonsleep deprivation groups for different prepulse intensities. PPI% values are different in different prepulse intensities (p < .05) (not denoted). SVA does not have a significant effect on PPI% (p > .05). Error bars represent standard deviations. NSD: nonsleep deprivation, SVA: sodium valproate, PPI: prepulse inhibition, dB: decibel.

**Figure 2:**
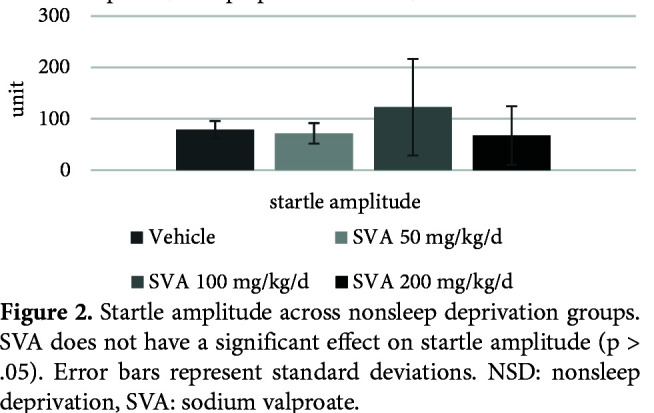
Startle amplitude across nonsleep deprivation groups. SVA does not have a significant effect on startle amplitude (p > .05). Error bars represent standard deviations. NSD: nonsleep deprivation, SVA: sodium valproate.

**Figure 3:**
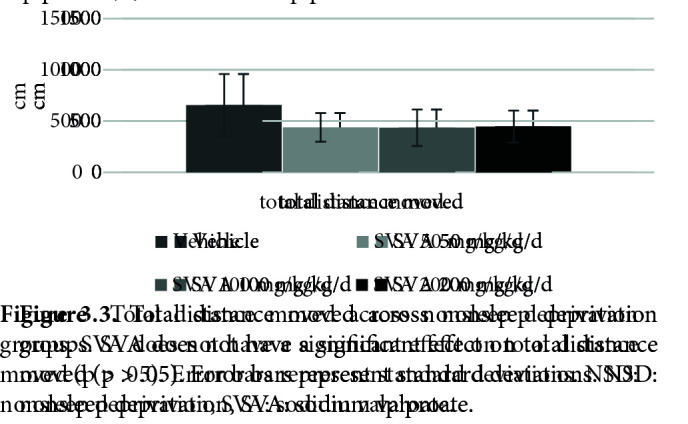
Total distance moved across nonsleep deprivation groups. SVA does not have a significant effect on total distance moved (p > .05). Error bars represent standard deviations. NSD: nonsleep deprivation, SVA: sodium valproate.

Figure 1 shows the effects of SVA on PPI% with 74 dB, 78 dB, and 86 dB prepulses, respectively. The nparLD F1F1 test revealed a significant main effect of prepulse intensity on PPI% [ATS (1.72) = 101.00, p = .00 < .05], a nonsignificant main effect of SVA on PPI% [ATS (2.83) = 1.30, p = .27 > .05] and a nonsignificant effect of interaction [ATS (4.71) = .44, p = .80 > .05]. There was no significant effect of SVA in the NSD groups on the startle values [X2 (3) = 6.32, p = .09 > .05] (Figure 2) or the total distance moved (LMA) values [X2 (3) = 3.46, p = .32 > .05] (Figure 3). 

### 3.2. SD effects 

**Figure 4:**
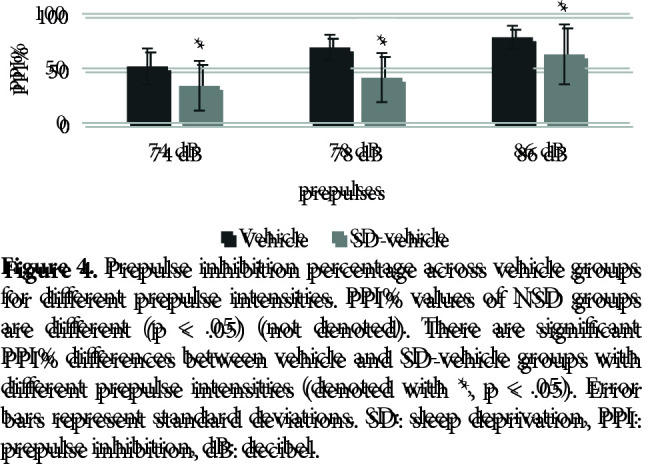
Prepulse inhibition percentage across vehicle groups for different prepulse intensities. PPI% values of NSD groups are different (p < .05) (not denoted). There are significant PPI% differences between vehicle and SD-vehicle groups with different prepulse intensities (denoted with *, p < .05). Error bars represent standard deviations. SD: sleep deprivation, PPI: prepulse inhibition, dB: decibel.

**Figure 5:**
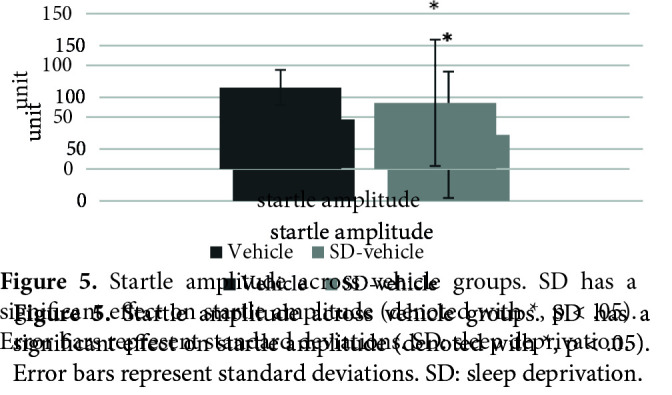
Startle amplitude across vehicle groups. SD has a significant effect on startle amplitude (denoted with *, p < .05). Error bars represent standard deviations. SD: sleep deprivation.

**Figure 6:**
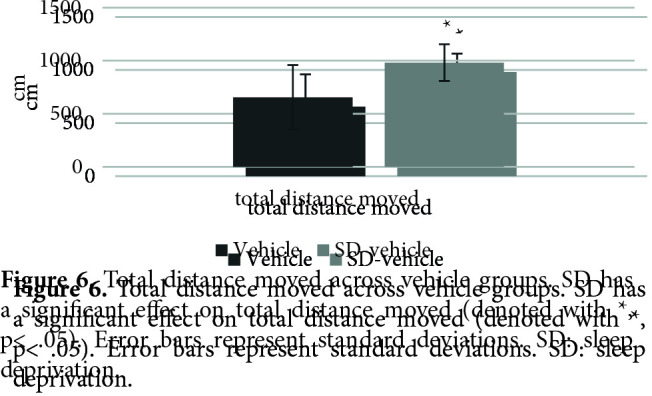
Total distance moved across vehicle groups. SD has a significant effect on total distance moved (denoted with *, p < .05). Error bars represent standard deviations. SD: sleep deprivation.

Figure 4 shows the effects of SD on PPI% with 74 dB, 78 dB, and 86 dB prepulses, respectively. The nparLD F1F1 test revealed a significant main effect of prepulse intensity on PPI% [ATS (1.91) = 34.23, p = .00 < .05], a significant effect of SD on PPI% [ATS (1.00) = 5.61, p = .01 < .05] and a nonsignificant effect of interaction [ATS (1.91) = 2.48, p = .08 > .05]. There was a significant difference between the startle values of the SD vehicle (mdn = 44.90) and vehicle (mdn = 84.25) groups [U = 11, z = –2.21, p = .02 < .05] (Figure 5). There was a significant difference between the total distance moved values of the SD vehicle (mdn = 977.86) and vehicle (mdn = 683.31) groups [U = 7, z = –2.63, p = .00 < .05] (Figure 6).

### 3.3. SVA effects in SD groups

**Figure 7:**
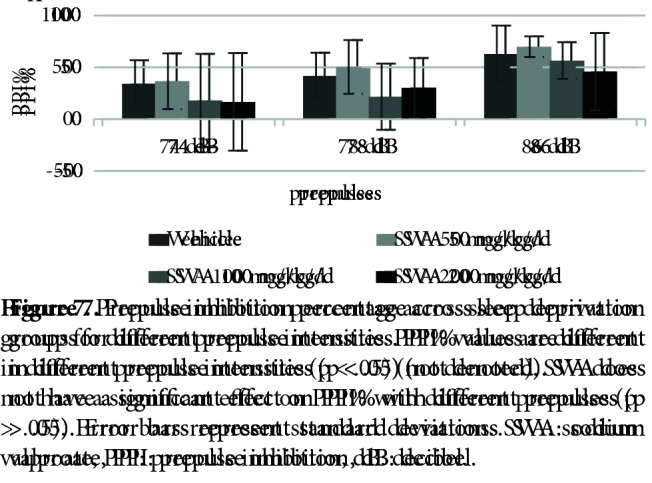
Prepulse inhibition percentage across sleep deprivation groups for different prepulse intensities. PPI% values are different in different prepulse intensities (p < .05) (not denoted). SVA does not have a significant effect on PPI% with different prepulses (p > .05). Error bars represent standard deviations. SVA: sodium valproate, PPI: prepulse inhibition, dB: decibel.

**Figure 8:**
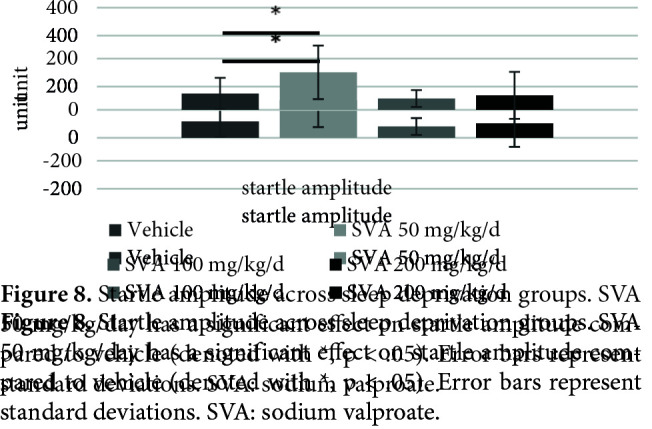
Startle amplitude across sleep deprivation groups. SVA 50 mg/kg/day has a significant effect on startle amplitude compared to vehicle (denoted with *, p < .05). Error bars represent standard deviations. SVA: sodium valproate.

**Figure 9:**
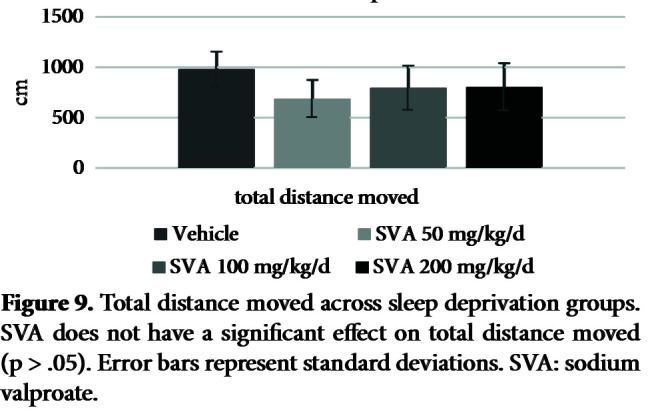
Total distance moved across sleep deprivation groups. SVA does not have a significant effect on total distance moved (p > .05). Error bars represent standard deviations. SVA: sodium valproate.

Figure 7 shows the effects of SVA in the SD groups on PPI% with 74 dB, 78 dB, and 86 dB prepulses, respectively. The nparLD F1F1 test revealed a significant main effect of prepulse intensity on PPI% [ATS (1.96) = 35.64, p = .00 < .05], a nonsignificant effect of SVA on PPI% [ATS (2.91) = 1.16, p = .32 > .05] and a nonsignificant effect of interaction [ATS (5.01) = .59, p = .70 > .05]. There was a significant effect of SVA in the SD groups on the startle values [X2 (3) = 9.20, p = .02 < .05] (Figure 8). The Mann–Whitney U test revealed a significant difference between vehicle (mdn = 44.90) and SVA 50 mg/kg/day group (mdn = 119.90) [U = 13.00, z = –2.00, p = .04 < .05]. There was also a nonsignificant effect of SVA on the total distance moved values [X2 (3) = 7.59, p = .055 > .05] (Figure 9).

## 4. Discussion

This study firstly showed that SVA did not have a statistically significant effect on the PPI%, startle, and LMA in the rats at the subchronic intraperitoneal doses of 50–100 and 200 mg/kg/day. Secondly, it was shown that 72-h SD significantly disrupted the PPI, decreased the startle, but increased the LMA in the rats. Afterward, it was determined that the SVA application at the same doses did not have a statistically significant effect on the disrupted PPI and increased LMA by SD. On the other hand, SVA 50 mg/kg/day administration after SD significantly increased startle amplitude. 

The main treatment of the condition of schizophrenia is achieved by antipsychotic drugs, while polypharmacy practices where MSs are added to existing antipsychotic treatments are very common. As there are few data supporting SVA usage in schizophrenia treatment, the drug usage is off-label, but its usage for this indication is prevalent [39]. According to studies, the frequency of adding SVA to schizophrenia treatment varies in the range of 15%–50% [40]. The reason for using SVA in schizophrenia patients is frequently associated with achieving impulse control and reducing aggression in patients that do not respond to classical treatment [40]. There are studies suggesting that SVA reduces aggression in schizophrenia patients. For example, it was shown that SVA reduces aggression in schizophrenia patients receiving risperidone or olanzapine treatment [41]. The effectiveness of SVA, which is a GABAergic drug, in schizophrenia, which is a disorder that is considered to be characterized by mesolimbic dopamine increase, is associated with its reduction of dopaminergic activity [42].

As known, PPI is disrupted in several psychiatric disorders, especially schizophrenia. It is accepted that drugs that are shown to reverse this disruption have an antipsychotic potential. In addition, little is known about the action mechanisms of MSs on PPI. A study conducted in 2006 by Umeda et al. [43] investigated the effects of MSs on PPI disrupted by apomorphine and MK-801 in mice. Accordingly, it was seen that valproate (30–300 mg/kg, i.p.) did not have any effect on PPI by itself, but it reversed the PPI disruption caused by apomorphine (1 mg/kg, s.c.), and as it did not induce any effect on the PPI disrupted by dizocilpine (0.3 mg/kg, s.c.), it was thought that it induces its effect through the dopaminergic system. Again, in rodent models, it was reported that valproate (100 mg/kg) did not affect PPI disruption induced by ketamine or amphetamine [44]. In an epigenetic schizophrenia mouse model induced by methionine, it was determined that valproate (1.5 mmol/kg) did not have an effect by itself on PPI, but when used together, it antagonized the reduction in PPI created by methionine [45]. 

Similar to the aforementioned studies, in this study, it was also shown that SVA did not have a significant effect on the PPI at the tested doses. However, although the difference was not statistically significant, SVA reduced the LMA at all doses that were used. It is expected that the LMA of rodents would decrease in the open field test in relation to anxiety [46]. Furthermore, many studies reported the anxiolytic properties of SVA [47,48]. For this reason, observation of a decrease in the LMA in this study by SVA was not an expected effect. However, it was considered that the known sedation effect of SVA [49] could have reduced the LMA. 

SD, which was applied at the second stage of the study, behaviorally induces a psychosis-like picture without any pharmacological intervention. It was reported that, in humans, anxiety, irritability, and perceptual distortions start by 24–48 h of SD, expected disorders and hallucinations are seen in 48–90 h, and delusions emerge after 72 h [50]. As this psychosis-like picture emerges after 48 h, in this study, the rats were subjected to SD for 72 h. The disruption of the sensorimotor gating and presence of hyperactivity in the rats after SD indicate that the model may be used as a psychosis model in experimental animals. 

It was observed that SVA, which started to be applied 24 h before 72-h SD and continued for a total of 4.5 days, did not create a statistically significant effect on the PPI% at doses of 50–100 and 200 mg/kg/d. However, although the difference was not significant, the 50 mg/kg/day form of SVA increased the already reduced PPI% at all prepulses, while the other doses appeared to reduce it even more. Likewise, at the applied doses, while SVA did not have a statistically significant effect on LMA, it appeared to reduce the LMA increased by SD (p = .057). At this point, SVA 50 mg/kg/day was the only dose reducing the LMA most, and it also significantly normalized the decreased startle amplitude levels by SD.

Considering animal models, the positive effects of SVA on sensorimotor gating are reported. In a schizophrenia mouse model, it was reported that SVA applied prophylactically for 14 days at 200 mg/kg 2×1 prevented PPI deficits and hyperactivity [51]. Another study showed that SVA acutely applied at 178–316 mg/kg increased the PPI% of mice with naturally low PPI [52]. Similarly, there are also studies and case reports suggesting that SVA reduces psychotic symptoms in schizophrenia when it is added to an ongoing antipsychotic treatment. It was reported that the addition of 1700 mg/day of SVA to the treatment of a patient with functional hallucinations receiving antipsychotic treatment improved hallucinations and noticeably increased the functionality of the patient [53]. A double-blind, randomized study determined that the addition of SVA by about 2300 mg to the treatment of olanzapine or risperidone at acute exacerbation of schizophrenia achieved a reduction in psychotic symptoms by using PANSS and BPRS [54]. In an open-label study where SVA was added at a mean dose of 1600 mg/day to the treatments of 28 schizophrenia patients with long disease durations, it was reported that 9 of the patients responded well to the treatment, 12 responded partially, and 7 did not respond or responded negatively (n = 2) [55]. Furthermore, in a review containing randomized controlled studies, it was reported that there is only limited evidence that SVA added to antipsychotic treatment improves the general clinical response, especially aggression [56]. Likewise, in a detailed review containing several studies, it was reported that adding SVA to the treatment in schizophrenia has a very limited benefit [57]. On the other hand, to the best of our knowledge, there are no broad-scaled studies reporting on the worsening effect of SVA on psychotic symptoms in psychotic patients. As in a study by Suzuki et al. [55], while it was noted that worsening was observed in schizophrenia patients who were given additional SVA treatment, diffuse negative effects were not mentioned. Probably for these reasons, the tendency of clinicians towards adding SVA to schizophrenia treatment is stronger.

On the other hand, low-dose SVA (500–1000 mg/day) is used in prophylaxis of migraine [58]. Action mechanisms of SVA include inhibition of voltage-dependent sodium channels and T-type calcium currents, potentiation of GABA concentrations via GABA-synthesizing and degrading enzymes, and modulation of the extracellular signal-regulated kinase pathway [59]. Consequently, it decreases neurogenic inflammation and prevents central sensitization that are results of cortical spreading depression, a phenomenon that has a prominent role in migraine pathophysiology [60]. 

In addition, it was showed that SVA prevents CSD-mediated activation of TRN [31], a GABAergic region within the thalamocortical circuit that has a role in sensory gating that is suggested to dysfunction in schizophrenia and migraine [5,31]. Hence, in considering the positive effects of low-dose SVA in both migraine treatment and sensorimotor gating, future studies may focus on revealing how SVA acts on particular brain regions related to sensorimotor gating. Moreover, low and high SVA were shown to have different pharmacological effects on the brain. It was reported that SVA affects GABA and aspartate metabolism at low doses, and as the dose rises, it begins to increase serotonin and other monoamine levels, decrease succinic semialdehyde dehydrogenase, decrease ATP levels, inhibit sodium currents, increase glutamate release, modulate calcium and potassium conductance, and inhibit GABA transaminase [61]. Therefore, it may be suggested that positive effects of low-dose SVA on sensorimotor gating following SD arise from its effects on GABA, aspartate and/or monoamines; nevertheless, dose-dependent effects of SVA, especially in brain regions involved in sensorimotor gating, should be investigated. 

While it is not possible to completely adapt the data obtained in this study to humans, the drug doses used in this study were based on the doses used in humans. In studies with experimental animals, to determine the corresponding dose of drugs in humans, the formula “Human equivalent dose (mg/kg) = Animal dose (mg/kg) × [Animal Km / Human Km]” is used [62]. Km is the correction factor, whereas it is found by dividing the average weight of the species (kg) by the body’s surface area (m2). Accordingly, the SVA doses used in this study corresponded to 567 mg/day, 1135 mg/day, and 2270 mg/day for a 60 kg person. While the doses of SVA used in humans are adjusted based on plasma levels, they frequently vary in 500–2500 mg. Therefore, in this study, SVA was used in a broad dose range to reflect the clinical usage of the drug substantially. 

This study had some limitations. The plasma levels of SVA were not checked in the study, and as it is a parenteral medication application, it was assumed that it showed the known pharmacodynamic and pharmacokinetic effects. Moreover, in a similar study conducted on rats before [63], the authors determined that the plasma drug (gabapentin) levels were halved after SD. As the pharmacokinetic properties of drugs may vary with the effect of SD, it is possible that SVA levels could also be changed, but this possibility was not examined. Additionally, the application of SVA for a longer time than that in this study may lead to the observation of different effects. Another limitation was that the study did not use a sham group where the effects of the water tank could be assessed. Nevertheless, in previous SD studies conducted at our laboratory, the sole effect of the water tank was examined by using a sham group, and it was observed that the sensorimotor gating in this group did not change, but LMA increased by a bit, while it was not as noticeable as the increase in the SD group. With the purpose of using a smaller number of subjects, reassessment of the sham effect was not considered necessary. The fact that two groups in the study included 7 rats rather than 8 was another limitation, and it may have affected the results.

This study where the effects of SVA on sensorimotor gating disrupted by SD were investigated is the first one in the literature to the best of our knowledge. In studies examining the effects of SVA, PPI was frequently disrupted by using pharmacological agents, and the effects of SVA on this disruption were then investigated. In this study, on the other hand, the behavioral symptoms of psychosis were induced via SD and without using a pharmacological method. Although projecting data obtained from experimental animals directly onto humans would not be convenient, this study suggested that, in psychotic pictures, sole medium-high dose SVA usage may affect sensorimotor gating negatively, while low-dose usage may have a positive effect. This situation may indicate that the drug has dose-dependent effects on sensorimotor gating. Again, the fact that SVA shows sedative effects in a broad dose range may explain its clinical activity reported in psychotic pictures. It seems necessary to investigate the benefits and risks of using SVA in psychotic pictures in more detail. On the other hand, in persons with sensorimotor gating disorders that do not show explicit psychotic symptoms, usage of SVA for other reasons may have a possibility of triggering psychotic symptoms. In this sense, it may be necessary for clinicians to more strictly monitor especially patients with a history of psychiatric disorders in their family.

## Informed consent

The study protocol was approved by the Local Ethics Committee of Üsküdar University on June 23, 2016 with the approval number of 2016-13.

## References

[ref1] (2010). Consequences of sleep deprivation. International Journal of Occupational Medicine and Environmental Health.

[ref2] (2014). Sleep deprivation disrupts prepulse inhibition and induces psychosis-like symptoms in healthy humans. Journal of Neuroscience.

[ref3] (1995). deprivation in the rat: an animal model of mania. European Neuropsychopharmacology.

[ref4] (2013). Decreased thalamocortical functional connectivity after 36 hours of total sleep deprivation: evidence from resting state FMRI. PLoS One.

[ref5] (2015). The role of the thalamus in schizophrenia from a neuroimaging perspective. Neuroscience and Biobehavioral Reviews.

[ref6] (2014). Sleep deprivation leads to a loss of functional connectivity in frontal brain regions. BMC Neuroscience.

[ref7] (1993). Cortical-striatal-thalamic circuits and brain glucose metabolic activity in 70 unmedicated male schizophrenic patients. American Journal of Psychiatry.

[ref8] (2004). Abnormal glucose metabolism in the mediodorsal nucleus of the thalamus in schizophrenia. American Journal of Psychiatry.

[ref9] (1991). The effect of sleep deprivation on cerebral glucose metabolic rate in normal humans assessed with positron emission tomography. Sleep.

[ref10] (2000). Neural basis of alertness and cognitive performance impairments during sleepiness. I. Effects of 24 h of sleep deprivation on waking human regional brain activity. Journal of Sleep Research.

[ref11] (2006). Frontal lobe metabolic decreases with sleep deprivation not totally reversed by recovery sleep. Neuropsychopharmacology.

[ref12] (2012). From revolution to evolution: the glutamate hypothesis of schizophrenia and its implication for treatment. Neuropsychopharmacology.

[ref13] (2005). Cortical inhibitory neurons and schizophrenia. Nature Reviews Neuroscience.

[ref14] (1996). Paradoxical sleep deprivation increases the content of glutamate and glutamine in rat cerebral cortex. Sleep.

[ref15] (2010). Pharmacological modulation of brain levels of glutamate and GABA in rats exposed to total sleep deprivation. Journal of Experimental Pharmacology.

[ref16] (2009). Luthi A. Consequences of sleep deprivation on neurotransmitter receptor expression. The European Journal of Neuroscience.

[ref17] (2003). Human sleep spindle characteristics after sleep deprivation. Clinical Neurophysiology.

[ref18] (2013). The effects of eszopiclone on sleep spindles and memory consolidation in schizophrenia: a randomized placebo-controlled trial. Sleep.

[ref19] (2014). Sleep spindle deficits in antipsychotic-naive early course schizophrenia and in non-psychotic first-degree relatives. Frontiers in Human Neuroscience.

[ref20] (2005). Reduced prepulse inhibition in unaffected siblings of schizophrenia patients. Psychophysiology.

[ref21] (2011). Tail-pinch stress and REM sleep deprivation differentially affect sensorimotor gating function in modafinil-treated rats. Behavioural Brain Research.

[ref22] (1974). The more or less startling effects of weak prestimulation. Presidential Address.

[ref23] (1999). Prepulse inhibition and habituation of the startle response are stable neurobiological measures in a normal male population. Biological Psychiatry.

[ref24] (2009). Top-down modulation of prepulse inhibition of the startle reflex in humans and rats. Neuroscience and Biobehavioral Reviews.

[ref25] (2008). Sleep deprivation disrupts prepulse inhibition of the startle reflex: reversal by antipsychotic drugs. The International Journal of Neuropsychopharmacology.

[ref26] (2018). and its role in primary headaches. The Journal of Headache and Pain.

[ref27] (2003). What kind of habituation is impaired in migraine patients. Cephalalgia.

[ref28] (2018). Somatosensory gating is altered and associated with migraine chronification: a magnetoencephalographic study. Cephalalgia.

[ref29] (2020). Acute sleep deprivation enhances susceptibility to the migraine substrate cortical spreading depolarization. The Journal of Headache and Pain.

[ref30] (1995). deprivation in the rat: an animal model of mania. European Neuropsychopharmacology.

[ref31] (2015). The thalamic reticular nucleus is activated by cortical spreading depression in freely moving rats: prevention by acute valproate administration. The European Journal of Neuroscience.

[ref32] (2003). Role of the amygdala in fear extinction measured with potentiated startle. Annals of the New York Academy of Sciences.

[ref33] (2006). The use of behavioral test batteries, II: effect of test interval. Physiology & Behavior.

[ref34] (2004). Sleep deprivation induced by the modified multiple platform technique: quantification of sleep loss and recovery. Brain Research.

[ref35] (2000). Social stability attenuates the stress in the modified multiple platform method for paradoxical sleep deprivation in the rat. Physiology & Behavior.

[ref36] (2013). Neurobiological consequences of sleep deprivation. Current Neuropharmacology.

[ref37] (2014). Effects of Wen Dan Tang on insomnia-related anxiety and levels of the brain-gut peptide ghrelin. Neural Regeneration Research.

[ref38] (2014). Effects of sleep deprivation on different phases of memory in the rat: dissociation between contextual and tone fear conditioning tasks. Frontiers in Behavioral Neuroscience.

[ref39] (1994). Use of mood stabilizers among patients with schizophrenia,. Psychiatric Services.

[ref40] (2014). Off-label use of sodium valproate for schizophrenia. PLoS One.

[ref41] (2004). Adjunctive divalproex and hostility among patients with schizophrenia receiving olanzapine or risperidone. Psychiatric Services.

[ref42] (2000). Randomized, placebo-controlled pilot study of divalproex sodium in the treatment of acute exacerbations of chronic schizophrenia. Journal of Clinical Psychopharmacology.

[ref43] (2006). Effects of mood stabilizers on the disruption of prepulse inhibition induced by apomorphine or dizocilpine in mice. European Journal of Pharmacology.

[ref44] (2005). An investigation of the efficacy of mood stabilizers in rodent models of prepulse inhibition. The Journal of Pharmacology and Experimental Therapeutics.

[ref45] (2005). Valproate corrects the schizophrenia-like epigenetic behavioral modifications induced by methionine in mice. Biological Psychiatry.

[ref46] (2013). Determination of motor activity and anxiety-related behaviour in rodents: methodological aspects and role of nitric oxide. Interdisciplinary Toxicology.

[ref47] (2018). Effect of valproate and pregabalin on human anxiety-like behaviour in a randomised controlled trial. Translational Psychiatry.

[ref48] (1992). Effect of sodium valproate on the open-field behavior of rats. Brazilian Journal of Medical and Biological Research.

[ref49] (2005). Psychotropic effects of antiepileptic drugs. Epilepsy Currents.

[ref50] (2018). Severe sleep deprivation causes hallucinations and a gradual progression toward psychosis with increasing time awake. Frontiers in Psychiatry.

[ref51] (2012). Prophylactic valproic acid treatment prevents schizophrenia-related behaviour in Disc1-L100P mutant mice. PLoS One.

[ref52] (2009). Mood stabilizers increase prepulse inhibition in DBA/2NCrl mice. Psychopharmacology.

[ref53] (2012). Functional hallucinations in schizophrenia responding to adjunctive sodium valproate. Indian Journal of Psychological Medicine.

[ref54] (2003). Effect of divalproex combined with olanzapine or risperidone in patients with an acute exacerbation of schizophrenia. Neuropsychopharmacology.

[ref55] (2009). Augmentation of atypical antipsychotics with valproic acid. An open-label study for most difficult patients with schizophrenia. Human Psychopharmacology Clinical and Experimental.

[ref56] (2016). Valproate for schizophrenia. The Cochrane Database of Systematic Reviews.

[ref57] (2009). Adjunctive lithium and anticonvulsants for the treatment of schizophrenia: what is the evidence?. Expert Review of Neurotherapeutics.

[ref58] (2002). A randomized trial of divalproex sodium extended-release tablets in migraine prophylaxis. Neurology.

[ref59] (2010). Handbook of Clinical Neurology.

[ref60] (2019). Current status of antiepileptic drugs as preventive migraine therapy. Current Treatment Options in Neurology.

[ref61] (2003). Valproate: past, present, and future. CNS Drug Reviews.

[ref62] (2016). A simple practice guide for dose conversion between animals and human. Journal of Basic and Clinical Pharmacy.

[ref63] (2020). Effect of gabapentin on sleep-deprivation-induced disruption of prepulse inhibition. Psychopharmacology.

